# The Role of B Cells in PE Pathophysiology: A Potential Target for Perinatal Cell-Based Therapy?

**DOI:** 10.3390/ijms22073405

**Published:** 2021-03-26

**Authors:** Marta Magatti, Alice Masserdotti, Anna Cargnoni, Andrea Papait, Francesca Romana Stefani, Antonietta Rosa Silini, Ornella Parolini

**Affiliations:** 1 Centro di Ricerca E. Menni, Fondazione Poliambulanza Istituto Ospedaliero, 25124 Brescia, Italy; marta.magatti@poliambulanza.it (M.M.); anna.cargnoni@poliambulanza.it (A.C.); andrea.papait@poliambulanza.it (A.P.); francesca.stefani@poliambulanza.it (F.R.S.); antonietta.silini@poliambulanza.it (A.R.S.); 2 Department of Life Science and Public Health, Università Cattolica del Sacro Cuore Facoltà di Medicina e Chirurgia, 00168 Roma, Italy; alice.masserdotti@unicatt.it; 3 Fondazione Policlinico Universitario “Agostino Gemelli” IRCCS, 00168 Roma, Italy

**Keywords:** perinatal cells, preeclampsia, B cells, placenta, amnion, umbilical cord, autoantibodies

## Abstract

The pathophysiology of preeclampsia (PE) is poorly understood; however, there is a large body of evidence that suggests a role of immune cells in the development of PE. Amongst these, B cells are a dominant element in the pathogenesis of PE, and they have been shown to play an important role in various immune-mediated diseases, both as pro-inflammatory and regulatory cells. Perinatal cells are defined as cells from birth-associated tissues isolated from term placentas and fetal annexes and more specifically from the amniotic membrane, chorionic membrane, chorionic villi, umbilical cord (including Wharton’s jelly), the basal plate, and the amniotic fluid. They have drawn particular attention in recent years due to their ability to modulate several aspects of immunity, making them promising candidates for the prevention and treatment of various immune-mediated diseases. In this review we describe main findings regarding the multifaceted in vitro and in vivo immunomodulatory properties of perinatal cells, with a focus on B lymphocytes. Indeed, we discuss evidence on the ability of perinatal cells to inhibit B cell proliferation, impair B cell differentiation, and promote regulatory B cell formation. Therefore, the findings discussed herein unveil the possibility to modulate B cell activation and function by exploiting perinatal immunomodulatory properties, thus possibly representing a novel therapeutic strategy in PE.

## 1. Introduction

Preeclampsia (PE) is a hypertensive disorder during pregnancy that affects 2% to 8% of all pregnant women and is one of the main causes of maternal and fetal morbidity and mortality worldwide [1,2]. The pathophysiology of PE is poorly understood; however, several lines of evidence support a role of the immune system in the development of PE. Among immune cells, B cells represent a dominant component in the pathogenesis of PE. Therefore, studies aimed to dissect the different B cell subsets and their number alterations could be of great importance in understanding the pathophysiology of PE. On the other hand, mechanisms that affect B cells and their activation status may represent a valid therapeutic approach in PE.

Perinatal cells are defined as cells from birth-associated tissues isolated from term placentas and fetal annexes and more specifically from the amniotic membrane, chorionic membrane, chorionic villi, umbilical cord (including Wharton’s jelly), the basal plate, and the amniotic fluid [3]. They constitute a promising tool as a therapeutic approach in PE, especially in the light of the recent identification of B cells as target of the immune modulatory action of perinatal cells.

In this review we summarize (i) the pathophysiology of PE, (ii) the role of immune cells in PE, (iii) with a focus on the role of B cells in PE. Moreover, we discuss the current knowledge about the (iv) in vitro and (v) in vivo effects of perinatal cells on B cells and (vi) how these effects could contribute to the development of a PE treatment.

## 2. Preeclampsia: Pathogenic Mechanisms

### 2.1. Definition and Pathogenesis

PE is defined by the presence of hypertension accompanied by proteinuria after 20 weeks of gestation or [1,2], in the absence of proteinuria, hypertension accompanied by the presence of severe features such as thrombocytopenia, renal insufficiency, impaired liver function, pulmonary edema, neurological signs, and fetal growth restriction [1,2,4]. The pathophysiology of PE is poorly understood, and the underlying mechanisms contributing to PE are an active area of research. What is clear is that PE is a placental disease, as the clinical syndrome does not develop in the absence of placenta, and delivery is the definitive treatment.

PE begins with abnormal placentation in the first trimester and is characterized by impaired trophoblast invasion and incomplete spiral artery remodeling, followed by an uteroplacental ischemia that drives the hypertensive, multi-organ failure response detected in the maternal preeclamptic syndrome [5]. The early phases of a normal implantation are characterized by a low oxygen tension environment, and this condition favors the proliferation of trophoblasts, which anchor the blastocyst to maternal decidua with spiral arteries. Subsequently, intervillous spaces allow the arrival of maternal blood, which increases oxygen tension and promotes the trophoblast differentiation from a proliferative to an invasive phenotype, with invasion and remodeling of spiral arteries.

### 2.2. Hypoxia and Oxidative Stress

Women with PE have upregulation of placental hypoxia-inducible factor (HIF), a marker of cellular oxygen deprivation, and hypoxia-related gene signatures, strongly suggesting a role of hypoxia in the pathogenesis of PE. Indeed, hypoxia may cause the failure of trophoblastic differentiation from the proliferative to the invasive phenotype. Consequently, poor spiral artery invasion may cause intermittent hypoxia and reoxygenation and generate oxidative stress [6]. When compared with women with normal pregnancies, women with PE display increased placental mitochondrial activity and production of reactive oxygen species (ROS), mainly superoxide anion, and decreased expression of superoxide dismutase and glutathione peroxidase, antioxidant enzymes involved in ROS neutralization [7,8].

### 2.3. Angiogenic Factors

Hypoxia has been shown to upregulate expression and secretion of soluble fms-like tyrosine kinase-1 (sFlt-1) protein in primary trophoblast cultures from first-trimester placentas [9]. sFlt-1 may represent a central molecule in PE because although its serum level increases significantly in pregnancy, there is a further increase in PE. sFlt-1 acts as a decoy receptor sequestrating circulating vascular-endothelial growth factor (VEGF) and placental growth factor (PlGF), thereby reducing their availability for angiogenesis. Injection of sFlt-1 in rats induced hypertension, proteinuria, and histological changes consistent with PE [10]. Soluble endoglin (sEng), a transforming growth factor (TGF)-β inhibitor, is another antiangiogenic, placenta-derived protein extensively studied in PE and found to be increased in sera of preeclamptic women. sEng is a coreceptor that binds to and decreases the levels of TGF-β, which normally induces proliferation and migration of endothelial cells [1]. Therefore, sFlt-1 and sEng facilitate downstream endothelial dysfunction, a vasoconstrictive state, microemboli, and placental ischemia and are positively correlated with severity of disease [11,12].

### 2.4. Heme Oxygenase, Hydrogen Sulfide, and Nitric Oxide Pathways

Moreover, a proximal pathway of Flt-1 induction involves the heme oxygenase (HO) enzyme, whose inhibition resulted in defective trophoblast invasion in vitro [13]. The HO enzyme degrades heme and leads to the formation of carbon monoxide (CO), which acts as a vasodilator and reduces perfusion pressure in the placenta [14]. Expression of the HO enzyme is reduced in PE [15], therefore potentially contributing to the pathogenesis of PE. On the other hand, adenoviral overexpression of HO enzyme (HO-1) in endothelial cells from preeclamptic villous explants inhibits sFlt-1 and sEng release, providing evidence for a protective role of HO-1 in pregnancy [16]. Similarly, to CO, hydrogen sulfide (H_2_S) and nitric oxide (NO) molecules have been described in the pathogenesis of PE. They have vasodilatory and angiogenic properties and have been shown to be decreased in PE [16,17]. Moreover, H_2_S modulates the levels of sFlt-1 and sEng through a VEGF-dependent mechanism [18].

## 3. Role of Immune Cells in Preeclampsia

### 3.1. Immune Cells in Physiological Pregnancy

In addition to the above-described mechanisms, there are several lines of evidence supporting a role of the immune system in the development of PE [19,20]. During normal pregnancy, soon after implantation, the maternal–fetal interface contains an intensified immune infiltrate, such as natural killer (NK) cells (~70% of total decidua lymphocytes), followed by macrophages (~20%), T cells (~10–20%), and rare dendritic and B cells [21,22,23]. NK cells and macrophages play a fundamental role in this pro-inflammatory stage of pregnancy by producing chemokines and growth factors involved in blastocyst implantation, trophoblast invasion, neo-angiogenesis, and spiral artery remodeling. Subsequently, during the second trimester of pregnancy, a shift pivotal to fetal growth occurs whereby the balance moves from pro-inflammatory to anti-inflammatory milieu characterized by the presence of a T helper (Th)-2 response and macrophages with a M2-like profile [24]. These macrophages phagocytize dying cells during trophoblast invasion and placental growth, thus preventing the release of potentially immunogenic paternal antigens. They also interact with NK cells and induce the generation of regulatory T cells (Tregs). Tregs are key players in maintaining fetal–maternal tolerance, in fostering an anti-inflammatory environment [25,26], and in promoting fetal survival by avoiding the recognition of paternal semi-allogeneic tissues by the maternal immune system [26].

### 3.2. Immune Cells in PE

Disturbance of fetal–maternal tolerance and alteration in immune homeostasis is widely considered to be a dominant component in the pathogenesis of PE [27]. In fact, pregnant mice genetically deficient in NK cells display several PE-like features, such as endothelial cell damage, alteration in placental growth, and necrosis of decidua [28]. Moreover, pregnant women at high risk of developing PE possess decidual NK with impaired ability to chemoattract trophoblast cells and to induce trophoblast outgrowth from placental villous explants, leading to partial spiral artery transformation and poor placentation [29]. Additionally, the numbers of monocytes and macrophages are altered in preeclampsia [30,31], and a decreased number of classical monocytes is observed in women with PE, accompanied by an increased number of intermediate monocytes [32] or non-classical monocytes [33]. An exaggerated immune activation is another important feature described in PE, and activation of monocytes during PE could be induced by different factors, among which are the increased level of pro-inflammatory cytokines, such as TNFα, IL-1β, IL-18, and the decreased level of the anti-inflammatory cytokine IL-10 [34]. Different from normal pregnancies, women with PE present an increased number of M1 macrophages. These M1 activated macrophages may produce pro-inflammatory cytokines and affect spiral arteries remodeling, influencing placental blood circulation [34]. In addition, PE is characterized by a pro-inflammatory state with an imbalance of Th1 and Th2 cells and cytokines, in favor of a Th1 predominance. Indeed, a heightened level of pro-inflammatory cytokines has been detected in plasma [35,36] and placenta [37] of women with PE, as well as an altered cytokine production by monocytes, NK cells, and lymphocytes compared with normal pregnant women [38,39]. Increase of Th17 subpopulation has been described in PE, with a significant correlation between PE development and Th17-, IL-2-, and IFN-γ-producing T cells [40,41]. Moreover, upregulation of Th17 immunity decreased Treg immunity [42], and several reports indicated low amount and functionality of specific subsets of Treg cells in PE both at the systemic [41,43,44,45] and the local level, within the decidual tissue [45,46]. In addition, decidual IL-17 positive cells are intimately involved in neutrophil infiltration [47], and in fact, neutrophils progressively increase in pregnancy both in the maternal systemic circulation and within the decidua, and these numbers are even higher in PE [48,49]. In normal pregnancy, neutrophils induce protective immunity against extracellular microbes in the uterus, but an excess of activated neutrophils, as found in PE, may enhance the release of typical inflammatory mediators in PE, such as ROS, TNF-α, and myeloperoxidase (MPO), causing hypertension and endothelial dysfunction [50,51,52].

## 4. Focus on B Cells

### 4.1. B Cell Main Features

**Figure 1:**
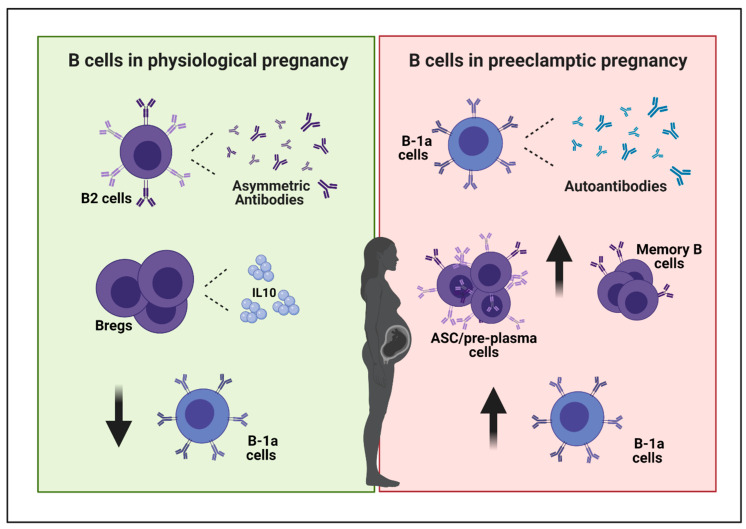
B cells and pregnancy. During physiological pregnancy B cells contribute to establish a tolerant environment. B2 cells generate asymmetric antibodies that bind paternal antigens but do not produce responses against them, protecting the fetus against a potential induction of the maternal lymphocyte response against paternal antigens. Moreover, regulatory B cells are found to be enhanced in healthy pregnancies, and they contribute to establishing a tolerant environment through the production of the anti-inflammatory IL-10. On the other hand, besides protective antibodies, the production of auto-antibodies (such as AT1-AA, antibodies against protein C and protein S) can be detrimental for pregnancy and characterize PE. A few indications suggest a role for B-1a cells in the production of these autoantibodies. A significant decrease in the frequency of B-1a cells has been reported in the peripheral blood of healthy pregnant women, while it has been reported to be elevated in patients with PE. Additionally, the percentage of memory B cells and plasma cell precursors increases in preeclamptic women. PE, preeclampsia; Bregs, regulatory B cells; IL-10, interleukin 10; ASC, antibody-secreting cells; AT1-AA, angiotensin receptor 1-autoantibodies. Created with BioRender.com (accessed on 21 February 2021).

Besides the immunological mediators described above, B cells also constitute a dominant element in the pathogenesis of PE [53] (Figure 1).

B cells are key components of the adaptive immune response and operate by producing antibodies as well as by regulating innate immunity by acting as antigen-presenting cells and producing cytokines.

Two major B cell populations have been described—namely, B1 and B2 cells. B1 cells derive during fetal and perinatal life from a precursor present in the fetal liver [54,55]. B1 cells are conventionally divided into B-1a and B-1b cells according to the expression of CD5 present on B-1a but not B-1b cells. In humans, in addition to CD5, the expression of CD27 and CD43 was recognized as being necessary for identifying B-1a cells [56]. In humans, B1 cells are primarily located in the peripheral blood, whereas in the murine system B1 cells mainly populate pleural and peritoneal spaces [57]. B-1b cells provide long-lasting immunity, producing adaptive antibodies upon antigen stimulation, whereas B-1a cells, in the absence of antigenic stimuli, produce natural antibodies, in particular IgM, which responds against a broad spectrum of infections [58].

Different from B1 cells, B2 cells originate in postnatal life and are produced from a committed pluripotent hematopoietic precursor (pro-B cell) present in the bone marrow (BM). Downstream, naïve B cells exit the BM and migrate to the follicles or the marginal zone of spleen for further maturation and differentiation into plasma and memory B cells. Follicular B cells and marginal zone B cells (MZ B cells) constitute the B2 cell population. Similar to B-1b and B-1a cells, the antigen exposure and signals from T helper cells activate and induce Igs class switching and B cell differentiation to follicular B cells, instead a T cell-independent activation, such as that mediated by Toll-like receptor stimulation, leads to the maturation and differentiation of MZ B cells [59,60,61].

### 4.2. B Cells in Physiological Pregnancy

During pregnancy B cells are careful coordinators of an immune response critical to the maintenance of feto–maternal tolerance. At first, they generate antibodies protecting the fetus against a potential induction of the maternal lymphocyte response against paternal antigens. Indeed, it was shown that maternal serum completely prevented the cytotoxic effect of maternal lymphocytes on cultured trophoblast, and the immunoglobulin IgG fraction was the major component responsible for this antigen-masking protective effect [62,63]. Some of these antigen-specific IgGs, called asymmetric antibodies, bind to paternal antigens with relative high affinity but lack the capacity to form antigen–antibody complexes and are incapable of activating immune effector functions, such as fix complement, phagocytosis, and cytotoxicity, thus allowing fetal survival [64]. Although not clearly established, different studies suggest that B2 cells might be the prime B cell subset responsible for the production of the pregnancy-protective asymmetric antibodies [65,66]. Moreover, regulatory B cells, a subtype of B cells with regulatory function, are found to be enhanced in the first trimester of healthy pregnancies, and therefore may contribute to establishing a tolerant environment in pregnancy by suppressing pro-inflammatory responses through the production of the anti-inflammatory IL-10 [67].

### 4.3. B Cells in PE

On the other hand, features of PE as an autoimmune condition are slowly being unveiled, and in this context B cells could participate in its pathogenesis by producing autoantibodies [53]. Indeed, in 1999, circulating autoantibodies to angiotensin receptor 1 (AT1) were reported in the sera of preeclamptic women [68]. AT1-autoantibodies (AT1-AA) are produced in response to placental ischemia and systemic inflammation [69] and mimic the natural ligand of the angiotensin type I receptor, highly expressed in the placenta, that harbors vasoconstrictive activity through the production of antiangiogenic factors s-Flt1 and endoglin [70], both hallmarks of the onset of the disease, as discussed above. Preclinical studies strongly confirmed the role of AT1-AA in the development of PE. Indeed, injection of AT1-AA isolated from preeclamptic women into pregnant mice induced hypertension, proteinuria, glomerular endotheliosis, placental abnormalities, and small fetus size, and these features were prevented by co-injection with an AT1 receptor antagonist [71]. Moreover, AT1-AA caused vasoconstriction in pregnant rats [72], increased lactate dehydrogenase release and caspase-3 and -8 activities in vitro in human umbilical cord vein endothelial cells [73], reduced immortalized human trophoblast cells invasion [74], and increased production of ROS in in vitro culture models of human vascular smooth muscle cells and trophoblast cells [75].

Besides AT1-AA, other autoantibodies were identified in PE patients and are suspected to account for the disease symptoms, such as antibodies against protein C and protein S or the thyroid autoantibodies [76,77]. Although the impact of autoantibodies in the development of PE has been very well described, the identification of a B cell subset responsible for their production has been reported only recently [78,79,80]. A few indications suggest a role for B-1a cells in the production of these autoantibodies. In fact, the frequency of CD19^+^CD5^+^ B-1a cells was reported to significantly decrease in the peripheral blood of healthy pregnant women during the third trimester [81,82], while it remained high in patients with PE [82,83]. Of note, the frequency of the total CD19^+^ B cells remains relatively constant in normal and in PE pregnancies [82]. Interestingly, Jensen et al. have demonstrated that lymphocytes of healthy pregnant women cultured in vitro in the presence of serum from PE patients greatly increased the percentage of CD19^+^CD5^+^ cells, very likely due to the high human chorionic gonadotropin levels present in the supernatant of serum and placenta of preeclamptic patients [82]. Moreover, CD19^+^CD5^+^ but not CD19^+^CD5^−^ cells isolated from peripheral blood of nonpregnant donors produce autoantibodies against AT1-receptor when cultured with serum from PE patients. In addition, in PE patients, CD19^+^CD5^+^ cells were detected in placental tissue, further reinforcing the idea of a role of B-1a cells and their polyreactive antibodies in the onset of PE [82].

Other changes of human circulating B cells that may contribute to the etiology of PE have been described. For example, the percentage of memory (CD27^+^CD38^−^) B cells and plasma cell precursors (CD27^+^CD38^+^) increased in preeclamptic women compared with the controls, and the percentages of plasma cells generated upon in vitro stimulation were significantly higher in the preeclamptic group than in the control group [84]. 

Given these premises, strategies affecting B cells and their activation status may represent a valid therapeutic approach in PE. Perinatal derivatives constitute a promising tool in this context, especially in the light of the recent identification of B cells as a target of their immune modulatory action [85,86]. The next paragraphs outline the state of the art of the crosstalk between perinatal derivatives and B cells and the potential for this interaction to be translated into a valid therapeutic option for PE.

## 5. B Cells as Target of Perinatal Cells

### 5.1. In Vitro

**Figure 2:**
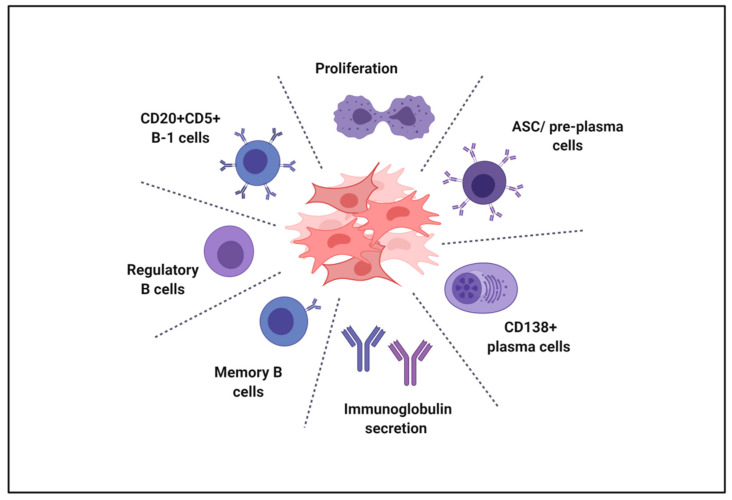
B cell targets of perinatal cells. In vitro and in vivo studies have shown that perinatal cells affect B cell proliferation and immunoglobulin secretion. Moreover, perinatal cells influence the differentiation of diverse B cell subsets (antibodies secreting cells, plasma cells, regulatory B cells, memory B cells, B-1a cells). ASC, antibody-secreting cells. Created with BioRender.com (accessed on 21 February 2021).

Cells isolated from perinatal tissues have drawn particular attention in recent years due to their ability to modulate several aspects of immunity, making them promising candidates for the prevention and treatment of various immune-mediated diseases [3]. As a matter of fact, numerous in vitro studies have widely demonstrated that cells from different perinatal tissues are able to interfere with the activation and the differentiation of cells belonging to both the innate and adaptive immune system, including macrophages, dendritic cells, natural killer cells, and T lymphocytes [87]. Although barely any knowledge is available regarding the effect of perinatal cells on B lymphocytes, some of the complex interactions between perinatal cells and B cells have been reported (Figure 2 and Table 1). The majority of the studies addressing the regulation of perinatal cells on B lymphocytes is limited to the description of a strong anti-proliferative capacity of perinatal cells on B cells, frequently associated with bioactive factors secreted by these cells rather than a direct cell–cell contact. For example, Che and colleagues co-cultured human mesenchymal stromal cells isolated from umbilical cord (hUC-MSC) with purified mouse splenic B cells and demonstrated, in a contact-independent setting, that hUC-MSC were able to abrogate the proliferation of activated B cells [88]. Similar observations were reported when human umbilical cord matrix cells were co-cultured with a B cell cancer line, the Burkitt’s lymphoma cell line [89], or with autoreactive B lymphocytes from peripheral blood mononuclear cells (PBMC) of immune thrombocytopenic patients [90].

These studies were supported by our group using other perinatal cells such as mesenchymal stromal cells (MSC) isolated from the amniotic membrane (hAMSC). Specifically, CD19^+^ B cell proliferation was suppressed in peripheral blood mononuclear cells (PBMC) co-cultured with conditioned medium obtained from the in vitro culture of hAMSC (CM-hAMSC), again underlining that cell-to-cell contact was not required. Similar results were obtained with B cells purified from peripheral blood, suggesting that hAMSC and their conditioned medium (CM) drive a direct inhibition on B cell proliferation without the involvement of other intermediary immune populations [85]. Human amniotic fluid stromal cells and their CM were also able to strongly inhibit B cell activation and proliferation, whereby they decreased the percentage of B cells in S phase cycle and significantly downregulated the expression of CD80/CD86 costimulatory molecules on activated B lymphocytes [91]. 

However, there are contrasting data on the inhibitory effect of perinatal cells on B cells. For example, human amniotic fluid stromal cells have been shown to inhibit the apoptosis of B lymphocytes, thus increasing activated B cell survival. In addition, human amniotic fluid stromal cells decreased the expression of the negative co-inhibitory molecules B7 homolog 4 (B7H4) and programmed death-ligand 1 (PD-L1) on activated B lymphocytes, molecules known to be involved in the negative control of inflammatory T cell (and possibly of B cell) responses [91]. Morandi and colleagues described an increase in B cell proliferation and a diminished spontaneous apoptosis in the presence of human amniotic epithelial cells (hAEC) [92]. Moreover, MSC isolated from umbilical cord were described to not affect or [93] dramatically support the in vitro growth of peripheral blood B cells, even in the absence of cell–cell contact [94].

As previously mentioned, B cell repertoire can be conventionally divided into two major populations, B1 and B2 cells [95,96], based on their phenotype, anatomic localization, self-renewing capacity, and production of natural antibodies. Interestingly, it was reported that perinatal cells can specifically impact the B1 cell subgroup (evaluated as CD20^+^CD5^+^) and in particular that human amniotic fluid stromal cells are able to downregulate the proportion of B1 cells [91], thus limiting the formation of the B cell subset mainly involved in the production of autoantibodies in PE.

CD19^+^ B cells can be further classified based on the differential expression of CD27 and CD38. Indeed, these two proteins are highly upregulated at late differentiation stages, therefore allowing the stratification of B cells into naïve/mature B cells (CD19^+^CD27^−^CD38^−^), memory B cells (CD19^+^CD27^+^CD38^−^), and antibody-secreting cells (ASC) or pre-plasma cells (CD19^+^CD27^+^CD38^+^). Within ASC, two subsets can be discriminated based on their expression of the proteoglycan CD138: plasmablasts (CD138^-^) and plasma cells (CD138^+^) [97]. ASC/pre-plasma cells CD27^+^CD38^+^ were found to be increased in PE women [84]. Different studies showed that the immunomodulatory capacity of perinatal cells and of their conditioned medium is linked to a blockage of ASC/pre-plasma cells CD27^+^CD38^+^ and linked also to the terminal differentiation of B cells into CD138^+^ plasma cells, consequently leading to a reduction in immunoglobulin secretion [85,88,91].

Contrasting results are reported for the activity of perinatal cells on memory B cells, another B cell subtype increased in PE [84]. Co-culture of purified B cells with human amniotic fluid stromal cells has been shown to be able to reduce the proportion of CD19^+^CD20^+^CD27^+^ memory B cells [91], while PBMC cultured in the presence of CM-hAMSC was shown to increase CD19^+^CD27^+^CD38^−^ memory B cells [85]. Although B cells may directly interact with perinatal cells, with a consequent alteration of their properties/function, the presence of other immune cells (such as in systems using PBMC instead purified B cells) may influence in a coordinated action the immune regulatory behavior of perinatal cells. In addition, different stimulation used to activate B cells and the lack of uniformity in the use of markers to characterize the B cell population could account for the distinct results observed in different experimental setups.

The generation of plasma cells relies on the repression of paired box 5 (PAX-5) and B cell CLL/lymphoma 6 (BCL-6) expression, keepers of B cell phenotypes [98], as well as on the strong expression of interferon regulatory factor 4 (IRF-4), PR/SET domain 1 (PRDM1, the gene encoding for the transcription factor B lymphocyte induced maturation protein-1, BLIMP-1), and X-box binding protein 1 (XBP-1), identified as hallmarks of differentiation to plasma cells [99,100]. To dissect the mechanisms through which perinatal cells could affect B cell maturation, recent observations on the effects of hAMSC and hUC-MSC on the expression of master regulators of CD138^+^ plasma cell formation were reported. Specifically, hUC-MSC were shown to increase PAX-5 and decrease PRDM1 expression on B cells [88], while CM-hAMSC strongly inhibited IRF-4, PRDM1, and XBP1 transcription, consequently impairing B cell terminal differentiation [85].

Perinatal cells not only modulate B cell function by influencing their differentiation toward plasma cells, but they also promote the formation of regulatory B cells (Bregs). Morandi and colleagues reported that hAEC promoted the expansion of CD19^+^CD24^hi^CD38^hi^ Bregs, inferring an involvement of the immunosuppressive molecule adenosine [92]. Xue Qun and colleagues, however, take the opposite view, suggesting that the immune modulation performed in vitro by perinatal cells might not directly involve IL-10^+^ Bregs, whose formation turns out to be inhibited by human amniotic fluid stromal cells [91]. 

Identification of the signaling pathways involved in B cell proliferation and differentiation that are affected by perinatal cells could help to partially clarify the impact of perinatal cells on B cells. We recently demonstrated the strong immunoregulation ability exerted by hAMSC on B cells activated in a T-independent manner through CpG oligodeoxynucleotides (CpG ODN) stimulus [85]. We show that two different signaling pathways activated downstream the CpG ODN stimulus: the Toll-like receptor 9 (TLR9)-myeloid differentiation primary response 88 (MyD88)-interleukin-1 receptor-associated kinase (IRAK)1/4 and TLR9-phosphatidylinositol 3-kinase (PI3K)-protein kinase B (AKT) pathways [101,102,103] are suppressed by hAMSC, causing a reduction of the expression of the CpG ODN-uptake sensors CD205, TLR9, and CD14. As a consequence, we detected the inhibition of IRAK-4 (intracellular signaling mediator) expression, followed by inhibition of mitogen-activated protein kinases (MAPK) (c-Jun N-terminal Kinase (JNK), p38 MAPK, extracellular signal-regulated kinase (ERK)) and nuclear factor kappa-light-chain-enhancer of activated B cells (NF-kB) pathways and a significant decrease in the expression of phosphorylated AKT [85]. Similar results have been described for hUC-MSC by Che N. and colleagues, who reported the inhibition of AKT and p38 MAPK phosphorylation in a dose-dependent manner in B cells activated in a T-dependent manner through B cell receptor (BCR) activation [88].

The exact mechanism by which perinatal tissue-derived cells regulate the immune response is still unknown, but a large body of evidence demonstrates that their restorative effect is largely mediated by the secretion of active molecules [104]. In literature, many soluble factors have been proposed as possible mediators of perinatal cell regulation of B cells. Previous data have demonstrated that prostanoids, and in particular prostaglandin E2 (PGE2), are partially involved in the mechanism through which perinatal cells exert their immunomodulatory effect on immune cells [105,106,107]. Two recent studies, including ours, have demonstrated that prostanoids may be partially involved in the immunomodulatory actions of perinatal cells on B cells, but they showed a partially opposite effect. On one hand, prostanoids secreted by hAMSC promote the inhibitory capacity of these cells on proliferation and formation of antibody secreting cell but not on terminal maturation into plasma cells [85]. On the other hand, PGE2, released by hUC-MSC, might cooperate with other hUC-MSC-derived soluble cytokines to promote the proliferation and differentiation of humoral immunity in vitro [94]. 

### 5.2. In Vivo

Due to their intriguing immunomodulatory properties, perinatal cells have shown therapeutic effects in the treatment of inflammatory and immune-mediated diseases, such as lung [108,109] and liver [110] fibrosis, inflammatory bowel disease [111], wound healing [107,112], collagen-induced arthritis [111], multiple sclerosis [111], traumatic brain injury [113], experimental autoimmune encephalomyelitis [111], cerebral ischemia [114], Huntington’s disease [115], and diabetes [116]. 

Very few studies explored and proved the therapeutic efficacy of perinatal cells in pathological conditions driven by B cells (Figure 2 and Table 1). 

Recently, our group provided key insights into the effect of hAMSC on B lymphocytes in a mouse model of idiopathic pulmonary fibrosis (IPF), a lung disease driven by inflammation and characterized by progression of fibrotic processes [86]. The pathogenesis of IPF remains poorly understood, but the presence of lymphoid aggregates, containing either T or B cells, in the lung tissue concurrent with autoantibodies to self-antigens in IPF patient serum suggests a breakdown in central tolerance in adaptive immune cells [117]. Treatment with hAMSC reduced fibrosis progression and better preserved alveolar integrity. In parallel, hAMSC treatment maintained low levels of B cells in alveolar spaces and reduced the amount of CD138^+^ antibody-secreting cells in lung tissues, suggesting a decrease in B cell recruitment and an impairment of the maturation of B cells. Moreover, hAMSC counteracted the formation and expansion of intrapulmonary lymphoid aggregates. The results of this study pointed at a role for hAMSC in reducing the progression of fibrotic lesions by modulating the lung expression of different homeostatic lymphoid chemokines, such as chemokine (C-X-C motif) ligand 13 (CXCL13), and the expression of B-cell activating factor (BAFF), involved in B cell survival and maturation [86]. 

In another study, allogenic transplantation of hAEC showed remarkable therapeutic effects in animal models of autoimmune disorders, such as Hashimoto’s thyroiditis (HT) and systemic lupus erythematosus (SLE) [118]. Despite being characterized by different target organs and inflammatory processes, both of these diseases develop as a result of the breakdown of the immune tolerance to self-antigens, characterized by presence of autoantibodies produced by activated B cells and self-reactive T cells that act against self-antigens, consequently resulting in tissue damage [119,120]. hAEC injection reduced the severity of the disease, with organ function preservation, reduction of inflammation, and restoration of immune-balance in experimental autoimmune thyroiditis (EAT) and SLE mice. Focusing on B cell compartment, a selective and significant upregulation of Bregs was reported in EAT mice upon hAEC treatment, laying out regulatory B cells that produce IL-10 (B10) as the major target subset of hAEC. Nevertheless, hAEC had little effect on the B10 population in SLE mice, where evidence of reduced autoantibody production was reported. These distinct results suggest a different immune modulatory mechanism of hAEC according to disease, probably related also to the distinct Bregs behavior in these autoimmune disorders [118].

The effect of perinatal cells on the B cell compartment was also evaluated in a clinical study in which hUC-MSC were infused as a strategy for chronic graft-versus-host disease (cGVHD) prophylaxis in patients after human leukocyte antigen (HLA)-haploidentical hematopoietic stem-cell transplantation (HLA-haplo HSCT) [121]. In recent years, in fact, remarkable advances support the idea that, although the T cells remain the main effectors, B cells may also play an important role in the pathogenesis of cGVHD. The mechanisms by which B cells contribute to cGVHD are still incompletely understood, but a marked perturbation of B cell homeostasis was detected in cGVHD patients [122]. In particular, studies have shown that cGVHD patients exhibit a deficiency of memory B cell subpopulation and that this B lymphocyte subset positively impacts long-term allograft acceptance after renal transplantation [122,123]. The results of this clinical trial showed that repeated infusion of hUC-MSC might minimize the severity and the symptoms of cGVHD. Concurrently, changes in the numbers and subtypes of T, B, and NK cells were detected, suggesting a possible improvement of immune tolerance. Concerning B cells, a relevant increase of CD27^+^ memory B cells was observed after hUC-MSC injection, suggesting that perinatal cell infusion most likely affected the memory B cell subpopulation. 

**Table 1:** Effects of perinatal cells on B cells.

Perinatal Cells	Experimental Model	Effects on B Cells	Mechanisms Involved	Ref
**Human umbilical cord mesenchymal stromal cells**	In vitro co-cultured with purified mouse splenic B cells stimulated by CpG+ IgM + CD40L + IL-4 in a contact-independent setting	- Abrogation of B220^+^ B cell proliferation - Blockage of the terminal differentiation into CD138^+^ plasma cells - Reduction in immunoglobulin secretion	- Increase of PAX-5 and suppression of Blimp-1 mRNA expression - Inhibition of Akt and p38 MAPK phosphorylation	[88]
	In vitro co-cultured with the Burkitt’s lymphoma cell line, even in the absence of cell–cell contact	- Abrogation of lymphoma cells proliferation	- Arrest of lymphoma cells division in the S phase	[89]
	In vitro co-cultured with autoreactive B lymphocytes from peripheral blood of immune thrombocytopenic patients	- Abrogation of CD19^+^ B cell proliferation	- Not investigated	[90]
	In vitro co-cultured with human PBMC stimulated by PHA	- Not inhibition of CD19^+^ B cell proliferation	- Not investigated	[93]
	In vitro co-cultured with human purified B cells stimulated by CpG+ IgA + IgG + IgM + CD40L + IL-2, even in the absence of cell–cell contact	- Stimulation of CD19^+^ B cell proliferation - Increase of CD138^+^ cell terminal differentiation, and immunoglobulin production	- Prostaglandin E2	[94]
	Clinical trial of cGVHD prophylaxis in patients after HLA-haplo HSCT	- Increase of CD27^+^ memory B cells	- Not investigated	[121]
**Human amniotic membrane mesenchymal stromal cells**	In vitro co-cultured of human PBMC and purified B cells stimulated by CpG with conditioned medium from hAMSC	- Suppression of CD19^+^ B cell proliferation - Blockage of ASC/pre-plasma cells CD27^+^CD38^+^ formation - Blockage of the terminal differentiation into CD138+ plasma cells - Increase CD19^+^CD27^+^CD38^−^ memory B cells	- Inhibition of IRF-4, PRDM1, XBP1 mRNA expression - Suppression of TLR9-MyD88-IRAK1/4 and TLR9-PI3K-AKT signaling pathways - Reduction of CpG sensors (CD205, TLR9, and CD14) - Inhibition of IRAK-4, and phosphorylated MAPK (JNK, p38 MAPK, ERK), NF-kB, and AKT - Prostanoids may be involved	[85]
	Experimental mouse model of idiopathic pulmonary fibrosis	- Reduction of B220 B cells in alveolar spaces - Reduction of CD138^+^ antibody-secreting cells in lung tissues	- Down-modulation of CXCL13 and BAFF lung expression	[86]
**Human amniotic fluid stromal cells**	In vitro co-culture of human purified B cells stimulated by CpG+ Immunoglobulin + CD40L +IL-2 + IL-4	- Inhibition of CD19^+^ B cell proliferation and activation (CD80/CD86 expression) - Reduction of the proportion of CD19^+^CD20^+^CD27^+^ memory B cells - Blockage of CD19^+^CD20^−^CD27^+^ plasma cells - Reduction in immunoglobulin secretion - Inhibition of IL-10^+^ cells Breg formation - Downregulation of the proportion of CD5^+^ B1 cells - Inhibition of apoptosis of activated B cell	- Decrease in the percentage of B cells in S phase cycle - Downregulation of the expression of CD80/CD86 costimulatory molecules on activated B lymphocytes - Decrease in the expression of B7H4 and PD-L1 in activated B cells may be involved	[91]
**Human amniotic epithelial cells**	In vitro co-culture with human purified B cells from peripheral blood stimulated by CpG+ CD40L + IL-4	- Increase in B cell proliferation - Diminished spontaneous apoptosis - Expansion of CD19^+^CD24^hi^CD38^hi^ Bregs	- Adenosine may be involved	[92]
	Experimental mouse model of autoimmune thyroiditis	- Upregulation of IL-10^+^ Bregs	- Not investigated	[118]
	Experimental mouse model of systemic lupus erythematosus	- Reduction of autoantibody production	- Not investigated	[118]

CpG, Unmethylated cytosine-guanine (CpG) motif-containing oligodeoxynucleotides (ODNs); CD40L, CD40 ligand; PAX-5, paired box 5; BLIMP-1, B lymphocyte induced maturation protein-1; AKT, protein Kinase B; MAPK, mitogen-activated protein kinases; HLA-haplo HSCT, Human Leukocyte Antigen-haploidentical hematopoietic stem-cell transplantation; IRF-4, interferon regulatory factor 4; PRDM1, PR/SET domain 1; XBP-1, X-box binding protein 1; TLR9-MyD88-IRAK1/4, Toll-like receptor 9 (TLR9)-myeloid differentiation primary response 88 (MyD88)-interleukin-1 receptor-associated kinase (IRAK)1/4; TLR9-PI3K-AKT, TLR9-phosphatidylinositol 3-kinase (PI3K)-protein kinase B (AKT); ERK, extracellular signal-regulated kinase; JNK, c-Jun N-terminal Kinase; NF-kB nuclear factor kappa-light-chain-enhancer of activated B cells; PBMC, peripheral blood mononuclear cells; PHA, phytohemagglutinin; cGVHD, chronic graft-versus-host disease. 

## 6. Conclusions

While considering the very complex pathogenesis of PE, a growing body of evidence suggests an important role of the altered immune system in the development of this disorder. In particular, B cells represent a dominant component of PE, leading to consideration of PE as an autoimmune-like disease affecting the maternal–fetal interface, especially due to the presence of circulating autoantibodies in PE patients [68,69]. It is known that B cells play an important role in autoimmune and immune-mediated diseases, both as pro-inflammatory cells, functioning either as antigen-presenting cells and immunoglobulin-secreting cells, and regulatory cells, producing cytokines that control T cell, macrophage, and natural killer cell functions. 

Thus, the possibility of modulating B cell activation and function by exploiting perinatal immunomodulatory properties, could be a novel therapeutic tool, overcoming the limitations of current treatments for PE [53]. As a matter of fact, ongoing therapeutic approaches for B cell-mediated diseases are mainly based on global B cell depletion (with a strong impact on general immune response), while perinatal cell administration could be a strategy for a more refined modulation of B cell activity, focused on maintaining normal B cell function and eliminating pathogenic B cells, which could prove more beneficial for patients. 

Several studies described the capacity of perinatal cells to impact on B cell response at different levels: by inhibiting B cell proliferation, impairing B cell differentiation, and promoting B regulatory cell formation. It could be of great interest to understand whether the activities exerted by perinatal cells on B cells are guided by the surrounding pathological/inflammatory microenvironment and are therefore dependent on the specific context. 

However, although there is evidence of the ability of gestational tissue-derived cells to favorably interact with B cells, some studies, in contrast, have reported pro-inflammatory effects of perinatal cells on B cells. These conflicting results could be due to their application of different experimental setups, such as using variable co-culture systems, stimulating conditions, B cell lines, and perinatal cells from diverse sources.

In conclusion, after reviewing the literature, it is clear that perinatal cells hold the potential of being a novel therapeutic approach for PE. However, the contradictory results and the mainly small number of studies exploring the effect of perinatal cells on B cell compartment cannot allow one to draw an unequivocal conclusion. Further in vitro and in vivo studies are necessary to better elucidate the immunomodulatory capacity of perinatal cells as a viable strategy for the treatment of preeclampsia. Specifically, comparative studies using perinatal cells derived from different compartments of the placenta and fetal annexes could be of interest for understanding which cells better affect B cells. Moreover, while there is the evidence of the effect of perinatal cells on B cells from healthy donors, the investigation of B cells from PE pregnancy needs to be addressed, as well as the evaluation of the efficacy of perinatal cells in animal models of PE.

## Data Availability

No new data were created or analyzed in this study. Data sharing is not applicable to this article.

## Abbreviations

PE	Preeclampsia
HIF	Hypoxia-Inducible Factor
ROS	Reactive Oxygen Species
sFlt-1	soluble Fms-like tyrosine kinase-1
VEGF	Vascular-Endothelial Growth Factor
PlGF	Placental Growth Factor
sEng	Soluble Endoglin
TGF	Transforming Growth Factor
HO	Heme Oxygenase
CO	Carbon Monoxide
H_2_S	Hydrogen Sulfide
NO	Nitric Oxide
NK	Natural Killer
Th	T helper
Tregs	Regulatory T cells
TNF	Tumor Necrosis Factor
IFN	Interferon
MPO	Myeloperoxidase
hUC-MSC	Human Umbilical Cord Mesenchymal Stromal Cells
PBMC	Peripheral Blood Mononuclear Cells
hAMSC	Human Amniotic Mesenchymal Stromal Sells
CM	Conditioned Medium
B7H4	B7 homolog 4
PD-L1	Programmed Death-Ligand 1
hAEC	Human Amniotic Epithelial Cells
ASC	Antibody-Secreting Cells
PAX-5	Paired Box 5
BCL-6	B-cell CLL/Lymphoma 6
IRF4	Interferon Regulatory Factor 4
PRDM1	PR/SET Domain 1
BLIMP-1	B Lymphocyte Induced Maturation Protein-1
XBP-1	X-box Binding Protein 1
CpG ODN	CpG Oligodeoxynucleotides
TLR9	Toll-Like Receptor 9
MyD88	Myeloid Differentiation Primary Response 88
IRAK1	Interleukin-1 Receptor-Associated Kinase 1
IRAK4	Interleukin-1 Receptor-Associated Kinase 4
PI3K	Phosphatidylinositol 3-Kinase
AKT	Protein Kinase B
MAPK	Mitogen-Activated Protein Kinases
JNK	c-Jun N-terminal Kinase
p38 MAPK	p38 Mitogen-Activated Protein Kinases
ERK	Extracellular Signal-Regulated Kinase
NF-kB	Nuclear Factor Kappa-Light-Chain-Enhancer Of Activated B Cells
BCR	B Cell Receptor
PGE2	Prostaglandin E2
IPF	Idiopathic Pulmonary Fibrosis
CXCL13	Chemokine (C-X-C motif) Ligand 13
BAFF	B-Cell Activating Factor
HT	Hashimoto’s Thyroiditis
SLE	Systemic Lupus Erythematosus
Bregs	Regulatory B cells
B10	Regulatory B cells that produce IL-10
cGVHD	Chronic Graft-Versus-Host Disease
HLA	Human Leukocyte Antigen
HLA-haplo HSCT	HLA-Haploidentical Hematopoietic Stem-Cell Transplantation
